# Transoral intraarticular cage distraction and C-JAWS fixation for revision of basilar invagination with irreducible atlantoaxial dislocation

**DOI:** 10.1186/s12891-020-03792-3

**Published:** 2020-11-20

**Authors:** Xiaobao Zou, Binbin Wang, Haozhi Yang, Su Ge, Bieping Ouyang, Yuyue Chen, Ling Ni, Shuang Zhang, Hong Xia, Xiangyang Ma

**Affiliations:** 1 The First School of Clinical Medicine, Southern Medical University, No.1838 North of Guangzhou Road, Guangzhou, 510515 People’s Republic of China; 2 Department of Orthopedics, General Hospital of Southern Theatre Command of PLA, No.111 Liuhua Road, Guangzhou, 510010 People’s Republic of China

**Keywords:** Basilar invagination, Irreducible atlantoaxial dislocation, Transoral approach, Revision surgery

## Abstract

**Background:**

The revision surgery of basilar invagination (BI) with irreducible atlantoaxial dislocation (IAAD) after a previous occipitocervical fusion (OCF) is challenging. Transoral revision surgery has more advantages than a combined anterior and posterior approach in addressing this pathology. The C-JAWS is a cervical compressive staple that has been used in the lower cervical spine with many advantages. Up to now, there is no report on the application of C-JAWS in the atlantoaxial joint. We therefore present this report to investigate the clinical outcomes of transoral intraarticular cage distraction and C-JAWS fixation for revision of BI with IAAD.

**Methods:**

From June 2011 to June 2015, 9 patients with BI and IAAD were revised by this technique after previous posterior OCF in our department. Plain cervical radiographs, computed tomographic scans and magnetic resonance imaging were obtained pre- and postoperatively to assess the degree of atlantoaxial dislocation and compression of the cervical cord. The Japanese Orthopedic Association (JOA) score was used to evaluate the neurological function.

**Results:**

The revision surgeries were successfully performed in all patients. The average follow-up duration was 18.9 ± 7.3 months (range 9–30 months). The postoperative atlas-dens interval (ADI), cervicomedullary angle (CMA), distance between the top of the odontoid process and the Chamberlain line (CL) and JOA score were significantly improved in all patients (*P* < 0.05). Bony fusion was achieved after 3–9 months in all cases. No patients developed recurrent atlantoaxial instability.

**Conclusions:**

Transoral revision surgery by intraarticular cage distraction and C-JAWS fixation could provide a satisfactory outcome for BI with IAAD after a previous unsuccessful posterior operation.

## Background

Basilar invagination (BI) with atlantoaxial dislocation (AAD) usually causes severe neurological impairment because of the prolapse of the odontoid process into the foramen magnum and ventral compression of the cervical cord [1, 2]. Spinal cord decompression is the key for the improvement of neurological symptoms. Reduction of atlantoaxial joint, descent of the dens, fixation and fusion were common treatment for BI with AAD. Because of more technical difficulty of anterior approach, in clinical practice, a posterior-only occipitocervical fusion (OCF) procedure is frequently used to address BI with AAD regardless of the reducibility [3, 4]. Once a satisfactory reduction was failed after OCF, a subsequent revision surgery, which is usually requisite for the residual compression of the spinal cord, will be more difficult to perform because the posterior structure of the craniocervical level has been destroyed in previous OCF procedure [5, 6]. In addition, the contracture of the anterior muscles, ligaments, and capsules of atlantoaxial joint, and the formation of scar and osteophytes cause irreversible AAD, that makes reduction difficult. Transoral revision surgery has more advantages for such cases, but the familiar fixation device, like transoral atlantoaxial reduction plate (TARP), has a larger profile, that make the operation inconvenient and lead to more implants related complications [2, 5]. It has been reported that C-JAWS, a cervical compressive staple, can reduce the postoperative complications linked to the use of plates in anterior cervical discectomy and fusion (ACDF) due to its smaller profile [7, 8]. Until now, no reports on the use of C-JAWS for atlantoaxial fixation has been published.

In this study, a novel revision technique of transoral intraarticular cage distraction and C-JAWS fixation was performed in 9 cases of BI with irreducible atlantoaxial dislocation (IAAD) after a previous failed posterior OCF, and clinical data were retrospectively analyzed to evaluate the efficacy of this technique.

## Methods

### Patients

From June 2011 to June 2015, a total of 9 patients (4 men and 5 women, mean age 43 yrs., range 18–67) with BI with IAAD, who underwent a previous OCF but achieved unsatisfactory results, were treated by revision surgery through transoral intraarticular cage distraction and C-JAWS fixation in our department. Of nine cases, three patients with worsening of symptoms, and 6 with unchanged symptoms. The average interval between the first OCF and revision surgery was 84.7 ± 60.1 months (range 18–180 months) (see Table 1 for detailed information from the cases). The clinical presentations of the 9 subjects admitted to our hospital were as follows: occipital or neck pain (7/9, 77.8%), extremity numbness (8/9, 100%), extremity weakness (7/9, 88.9%), unsteady gait (3/9, 33.3%), and hemiparalysis (2/9, 22.2%) (Table 2).

**Table 1:** Demographic and clinical data of the 9 patients

Case	Age (year)/Sex	Anomalies in radiology	Previous surgery	Duration between the previous urgery and the revision (month)	Revision cause	Revision surgery
1	59/F	BI, IAAD, AA	OCF (C0–1)	120	IAAD with VSCC	PRI and TARF
2	41/M	BI, IAAD, AA, KFS, Chiari	OCF (C0–2)	36	IAAD with VSCC	PRI, PFD and TARF
3	57/M	BI, IAAD, AA	OCF (C0–3)	180	IAAD with VSCC	PRI and TARF
4	35/F	BI,IAAD, AA, KFS	OCF (C0–2)	65	IAAD with VSCC	PRI and TARF
5	67/F	BI, IAAD, AA, Chiari	OCF (C0–4)	47	IAAD with VSCC	PRI, PFD and TARF
6	27/M	BI, IAAD, AA, KFS, Chiari	OCF (C0–2)	132	IAAD with VSCC	PRI, PFD and TARF
7	36/F	BI, IAAD, AA	OCF (C0–2)	18	IAAD with VSCC	PRI and TARF
8	45/F	BI, IAAD	OCF (C0–3)	144	IAAD with VSCC	PRI and TARF
9	18/M	BI, IAAD, AA, KFS, Chiari	OCF (C0–1)	20	IAAD with VSCC	PRI and TARF

*BI* basilar invagination; *IAAD* irreducible atlantoaxial dislocation; *AA* atlas assimilation; *KFS* Klippel-Feil syndrome; *Chiari* Chiari malformation; *OCF* occipitocervical fusion; *PRI* posterior removal of instrument; *PFD* posterior fossa decompression; *VSCC* ventral spinal cord compression; *TARF* transoral atlantoaxial reduction and fixation

**Table 2:** Clinical symptoms

Symptoms	Preoperative no. (%)	Postoperative improvement no. (%)
Occipital or neck pain	7 (77.8%)	6 (85.7%)
Extremity numbness	8 (88.9%)	6 (75.0%)
Extremity weakness	7 (77.8%)	7 (100%)
Unsteady gait	3 (33.3%)	3 (100%)
Hemiparalysis	2 (22.2%)	2 (100%)

Each patient may have one or more symptoms

### Preoperative examinations

Before the revision surgery, cervical plain radiographs, low-dose biphasic computed tomography (CT) [9] scans and magnetic resonance imaging (MRI) were performed. All patients were found with BI and AAD on cervical radiographs and CT scans. There were 4 patients with C2–C3 fusion and Klippel-Feil syndrome (KFS), 8 cases with atlas assimilation and 3 subjects with Chiari malformation. Bony graft nununion was found on CT scans in all patients after first OCF. MRI showed obvious ventral spinal cord compression of the cervical cord in all of the cases. The average JOA score (17-point system) was 9.7 ± 2.2.

### Surgical procedure

For all 9 of the cases, posterior operations were performed to remove the previous implants of OCF before transoral revision surgeries. Posterior fossa decompression was additionally performed in 3 cases with Chiari malformation. All procedures were performed in one stage.

Preoperative preparations: An oral examination and dental cleaning were performed before surgery. Oral cleaning with 0.02% vinegar chlorhexidine was performed 3–6 times per day for 3 days before surgery. Broad-spectrum antibiotics were administered intravenously 30 min before surgery.

**Fig. 1:**
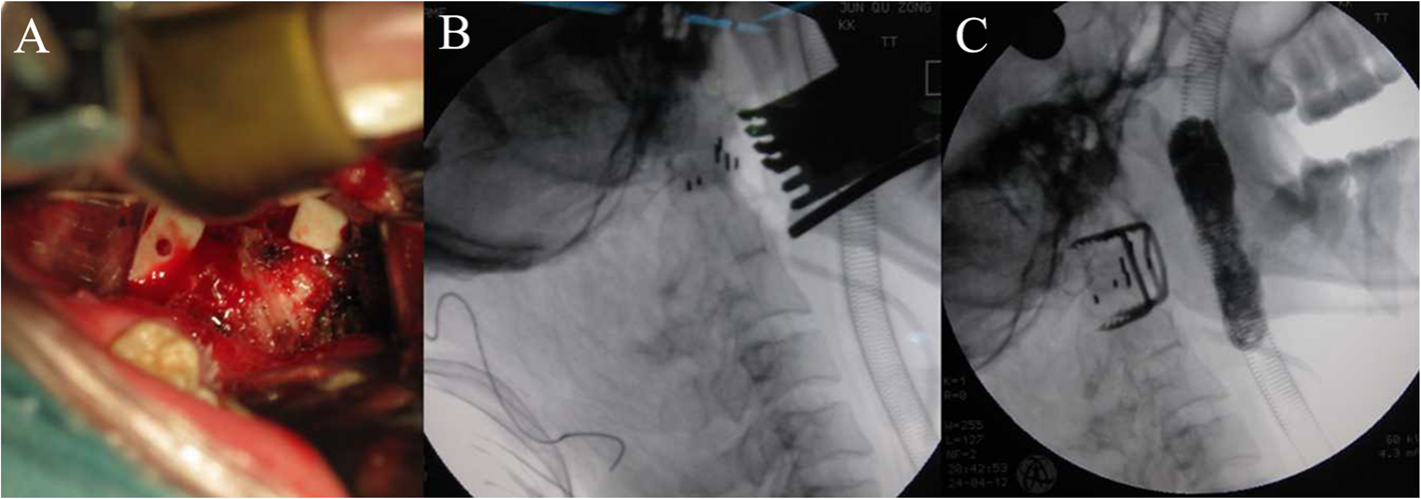
Intraoperative procedures. **a** Photograph after the placement of bilateral intraarticular cages. **b** Intraoperative X-ray after the implantation of bilateral intraarticular cages showed satisfactory atlantoaxial reduction. **C.** Intraoperative X-ray after the implantation of C-JAWS showed well position of devices

**Fig. 2:**
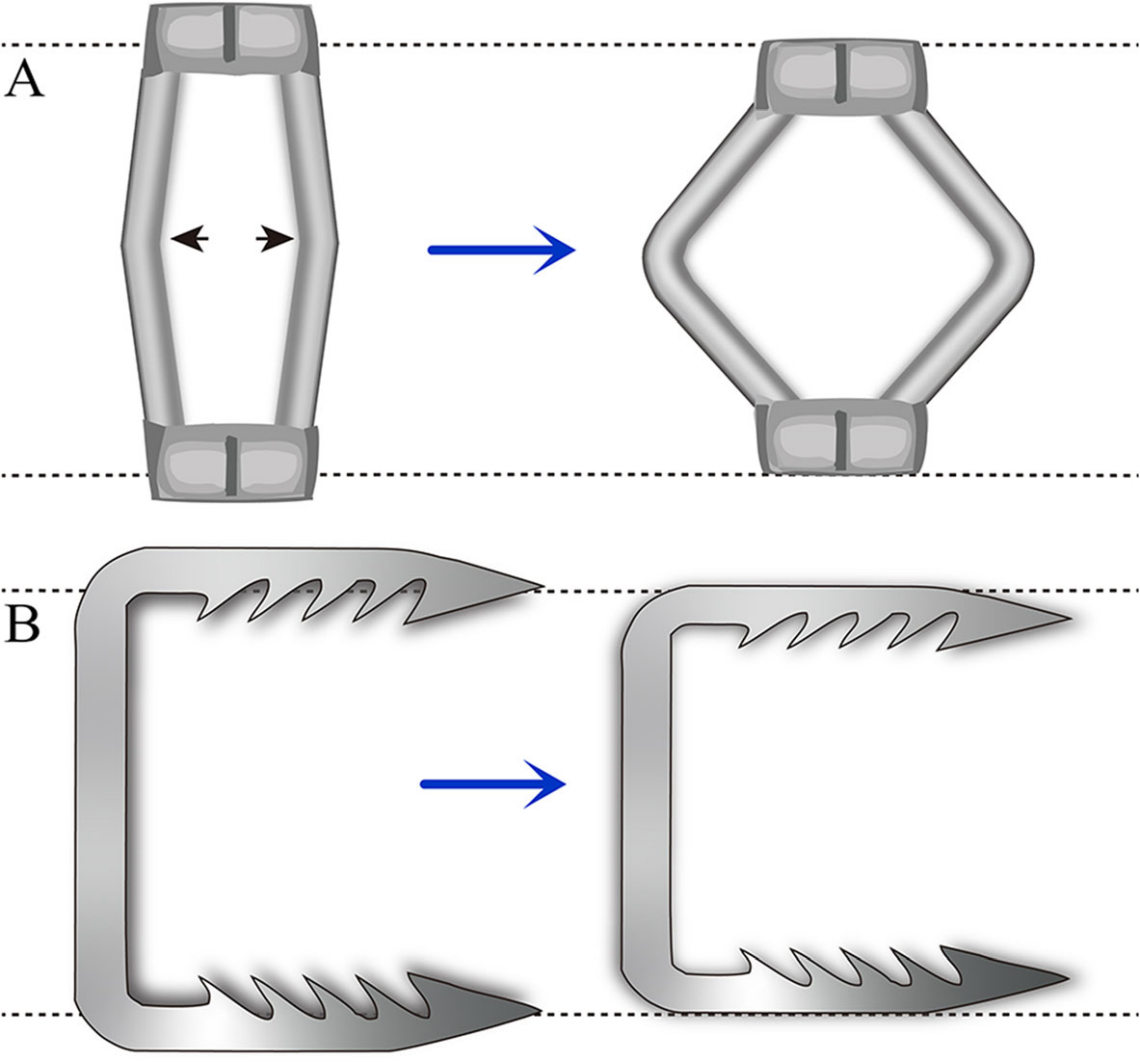
Operating mode of the C-JAWS. **a** On the frontal views, the bilateral arms of holder were distracted to shorten the distance between the top and bottom rivets to control compression. **b** The corresponding change of holder on lateral views

Surgical techniques: Under general anaesthesia with nasotracheal intubation, the patient was placed in prone position with skull traction of 4–12 kg. After posterior median longitudinal incising and stripping, the previous internal fixation was removed. Subsequently, the patient was changed to supine position with the same weight skull traction. After conventional oral cleaning and disinfection, a longitudinal incision sized 3–4 cm was made in the median posterior pharyngeal wall and the anterior structure of the C1–C2 was exposed after separating the mucosa and muscle. Then, the anterior scar tissue and hyperplastic callus between the odontoid and anterior arch of C1 were resected without removal of the odontoid. After the capsule of bilateral lateral mass joints was incised, the intraarticular adherent tissues and articular cartilage were removed with a curette and grinding drill. The bilateral lateral masses were then levered in the posterosuperior and anteroinferior directions, respectively, to completely loosen the atlantoaxial joint. Then, two appropriate intraarticular cages (Wego, Shangdong, China) filled with autologous iliac bone were put into the bilateral lateral mass joints for distraction and bony fusion (Fig. 1a). After reduction of the atlantoaxial joint was identified by intraoperative radiograph (Fig. 1b), holes were drilled at the midpoint of the lateral mass of C1 and the vertebral body of C2. The bilateral distances between the holes of C1 and C2 were measured and two appropriate-sized cervical compressive staples (named C-JAWS, Medicrea, Lyon, France) were chosen accordingly. Then, the C-JAWS was inserted into the desired position through preparative holes by slight knock. The bilateral arms of staples were then distracted to provide controlled compression on the joints to stabilize C1–C2 (Fig. 2). After a satisfactory reduction of the atlantoaxial joint and the position of C-JAWS was further confirmed by intraoperative radiograph (Fig. 1c), the muscular and mucosal were sutured in layers.

### Postoperative management and follow-up

The nasal trachea cannula was removed in 24–48 h postoperatively, and the nasogastric tube was removed on day 7 postoperatively. Ultrasonic nebulisation and 0.02% chlorhexidine acetate gargling were performed 3–6 times per day for 7 days. Broad-spectrum antibiotics were administered intravenously for 3 days. The X-ray, CT scan and MRI scan were performed postoperatively. The atlas-dens interval (ADI), the cervicomedullary angle (CMA), and the distance between the top of the odontoid process and the Chamberlain line (CL) were measured. The patients’ neurological status was assessed using the JOA scoring system. Bony fusion was confirmed by bony bridge formation on CT scan. All cases were required to wear a rigid cervical collar for 3 months after operation. All patients were followed up at 3, 6, 9 and 12 months and then once per year.

### Statistical analysis

SPSS 21.0 software (IBM, Armonk, NY, USA) was used for the statistical analysis. Measurement data were expressed as mean and standard deviation. ADI, CMA, CL and JOA scores before and after surgery were compared using paired-sample *t* test, and *P* value < 0.05 was considered statistically significant difference.

## Results

**Fig. 3:**
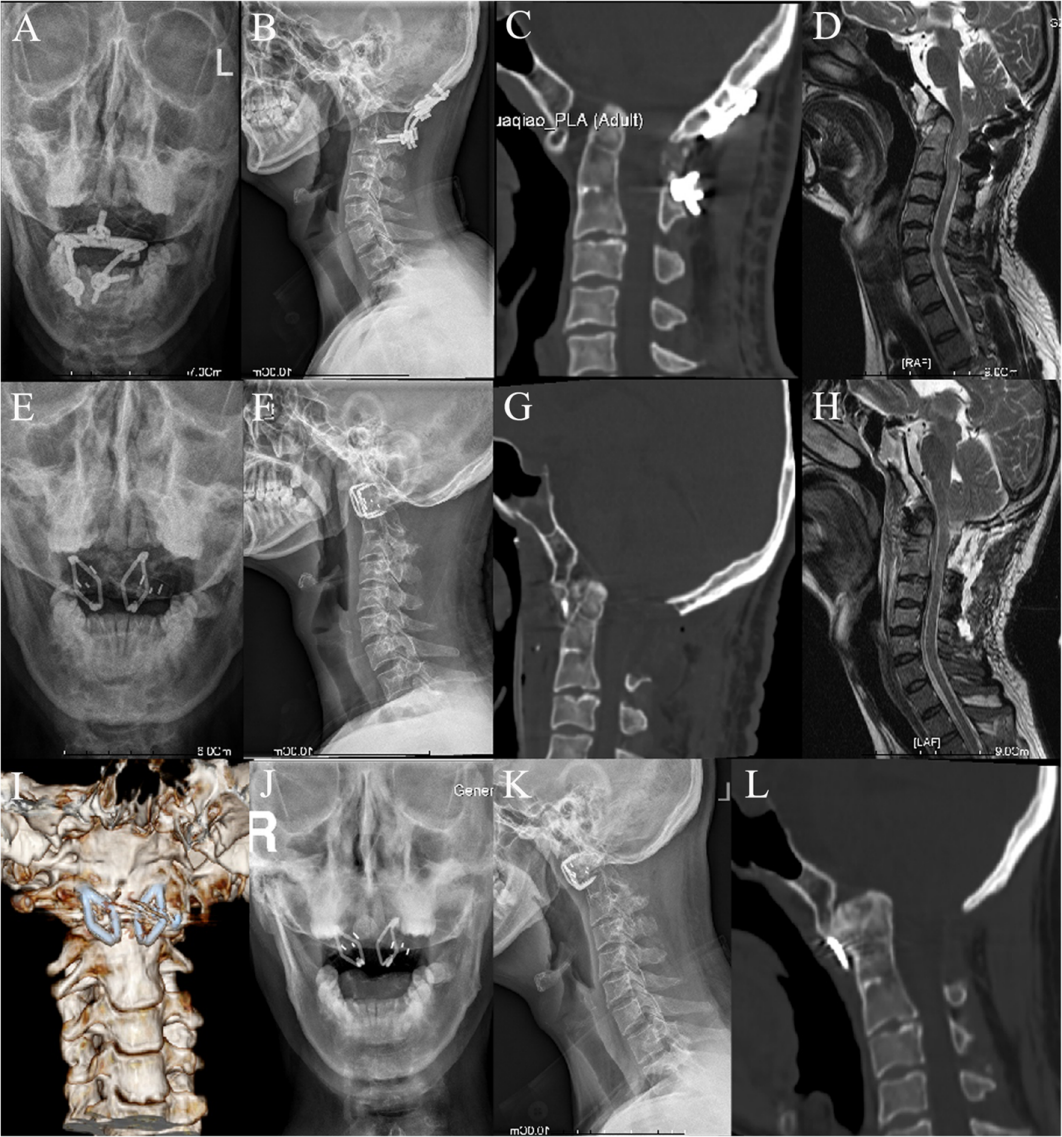
A 41-year-old man was diagnosed with BI with IAAD after a failed posterior OCF, and revised by transoral intraarticular cage distraction and C-JAWS fixation. **a-c** Images of cervical X-rays and CT scan before revision surgery showed evidence of BI with IAAD. **d** Preoperative Sagittal MRI showed compression of the cervicomedullary junction. **e-g** Postoperative cervical X-rays and CT scan performed at 1 week after revision surgery showed satisfactory reduction and fixation. **h** Postoperative sagittal MRI showed a desirable decompression of the cervicomedullary junction. **i** Postoperative three-dimensional reconstruction showed location of C-JAWS. **j-l** Cervical X-rays and CT scan at 6-month follow-up showed a solid bone fusion without loss of reduction

All the patients underwent transoral revision surgeries successfully after removal of the posterior internal fixation (Fig. 3). The average operative time was 251.1.9 ± 76.4 min (range 180–400 min), with average intraoperative blood loss of 170.6 ± 55.7 ml (range 90–250 ml). No spinal cord, vascular or dura mater injuries occurred during the operation. All patients obtained satisfactory reduction, good position of implants, decompression of spinal cord, and improvement of neurological function postoperatively. The average follow-up time was 18.9 ± 7.3 months (range 9–30 months). Clinical symptoms were relieved in all patients after revision surgery (Table 2). Significant improvements in ADI, CMA, CL and JOA were found at the 6-month follow-up visit (*P* < 0.05) (Table 3). All the cases obtained bony fusion in 3–9 months after the revision (Table 3). No complications occurred during the follow-up.

**Table 3:** Pre- and Postoperative data of the 9 patients

Case	JOA (preop)	JOA (postop)	ADI (preop)	ADI (postop)	CMA (preop)	CMA (postop)	CL (preop)	CL (postop)	Bone fusion confirmed (month)	Follow-up (month)	Complication
1	12	16	9.6	1.3	136.5	160.4	8.5	5.1	6	24	No
2	10	15	8.3	1.8	143.5	162.6	–	–	3	12	No
3	6	11	10.3	3.0	98.5	153.2	14.5	3.8	3	20	No
4	8	13	9.8	0	112.7	148.1	10.7	2.9	9	30	No
5	11	16	6.9	0	125.2	165.0	–	–	6	27	No
6	8	12	8.5	1.5	105.1	158.4	–	–	6	9	No
7	10	15	8.3	0	120.8	140.8	7.5	−0.5	3	12	No
8	13	17	7.1	0.6	133.8	170.1	15.6	3.0	9	21	No
9	9	13	8.7	1.0	110.3	139.2	12.7	0	3	15	No
M ± SD	9.7 ± 2.2	14.2 ± 2.0	8.6 ± 1.2	1.0 ± 1.0	120.7 ± 15.3	155.3 ± 10.8	11.6 ± 3.3	2.4 ± 2.2			
t	−25.931		21.243		−7.890		6.334				
p	0.000^a^		0.000^a^		0.000^a^		0.001^a^				

^a^Paired-sample t-test

*JOA* Japanese Orthopedic Association score; *ADI* atlas-dens interval; *CMA* cervicomedullary angle; *CL* distance between the top of the odontoid process and the Chamberlain line

## Discussion

BI, a relatively common malformation of the craniocervical junction, usually results in spinal cord and medulla compression if accompanied by AAD, which can cause severe neurological impairment and even sudden death [1–5]. It is usually diagnosed by cervical X-ray, CT and MRI. The osseous variation of the craniocervical junction can be revealed by X-ray and CT scan, and the spinal cord compression can be evaluated by MRI [10]. Diffusion tensor imaging can also be performed to identify the nature of the lesion if an unknown lesion is found in the vertebrae [11]. A surgical therapy is often necessary for this disorder to obtain symptoms alleviation and spinal cord decompression. It is generally believed that surgical approaches for such patients depend mainly on the reducibility of BI and AAD [12, 13]. If the BI and AAD can be reduced after axial cervical traction in hyperextended position, a posterior-only approach is indicated to achieve reduction, decompression, fixation and fusion [3, 14, 15]; If the BI or AAD cannot be reduced by cervical traction, a transoral anterior release and posterior reduction, decompression, fixation and fusion is often necessary [4, 16–18]. But, the high risks of catastrophic complications and technique demanding of anterior procedure make the posterior-only approach preferred for BI with IAAD in clinical practice. However, the decompression effect for a posterior-only procedure may be less desirable due to unsuccessful reduction. Subsequently, a revision surgery was required to promote neurologic recovery. If patients had achieved bony fusion after previous operations, transoral odontoidectomy could be used for decompression; If patients had not achieved bony fusion, successful reduction should be considered in revision surgery [5].

Routinely, a series of procedures including posterior removal of internal fixation, transoral anterior release and posterior fixation are required to achieve ideal reduction and decompression for cases who have already undergone a posterior OCF without bony fusion, if a posterior fixation is to be again adopted in revision surgery, which may cause tremendous trauma to patients. In order to judge the degree of the release more accurately and distract the atlantoaxial joint, the removal of posterior instruments should be performed before transoral anterior release. However, the change of position after transoral anterior release, which leads to an extremely unstability of atlantoaxial joint during operation, can also cause unexpected spinal injury [5]. Furthermore, the screw placement is difficult because the posterior anatomical structure of the craniocervical junction has been partly or completely destroyed after previous posterior fixation. Although the posterior screw insertion can be fulfilled by extending the segments of occipitocervical fusion as reported by Tan et al. [6] and Duan et al. [13], more cervical mobility is sacrificed.

Theoretically, the surgical related risks and injuries will significantly reduce if release, reduction, decompression, fixation and fusion can be performed through a transoral anterior-only approach after posterior removal of internal fixation. The transoral atlantoaxial reduction plate (TARP) system, designed by our institution in 2004, can achieve release, distraction, reduction, decompression, fixation and fusion in one stage with a transoral anterior-only approach [1, 5, 19–22], that provides an effective surgical choice for the treatment of BI with IAAD accompanied by ventral spinal cord compression. Yang et al. [5] presented 30 cases with BI and IAAD after posterior fossa decompression with or without fixation, and satisfactory decompressions and improvement of neurological functions with only loss of craniocervical junction mobility were obtained through transoral anterior revision surgeries using TARP system. Wu et al. [23] reported 1 patient of os odontoideum with IAAD treated by a previous failed posterior operation undergoing a successful transoral revision surgery using TARP system. But, according to our clinical experience, to conveniently accomplish the surgical procedures in a limited oral space, especially for patients with a small mouth is difficult for the high thickness and large shape of the TARP. Additionally, the requirement of sufficient soft tissues of the pharyngeal wall to cover the plate may lead to the occurrence of postoperative dysphagia and wound dehiscence [21, 24].

The C-JAWS, a cervical compressive staple, is commonly used in ACDF. Fiere et al. [7] reported a dependable biomechanical stability of the C-JAWS in a vitro testing, and the early clinical results of 23 cases who underwent ACDF using a C-JAWS with a thickness of 1.5 mm showed various advantages including short incision, short operative time and lower rate of dysphagia incidences as compared to most of the cervical anterior plate. Xia et al. [8] presented 9 cases undergoing ACDF with C-JAWS and a similar conclusion was concluded.

Because the C-JAWS is much thinner and smaller than the TARP, we deem it can also reduce complications associated with internal instruments, if it is applied in transoral atlantoaxial fixation. But the C-JAWS does not have the same function of joint distraction as TARP does, so an intraarticular cage was used in revision surgery for atlantoaxial joint distraction. In this study, 9 patients presenting with severe BI with IAAD had neurological function deterioration after a posterior OCF without bony fusion, and were revised by this novel transoral revision surgery. All patients achieved satisfactory reduction and significant improvement of neurological function without postoperative complications. According to our experience, this novel technique can facilitate the operation and reduce the complications associated with internal fixation. However, the C-JAWS only has a fixed function, and does not have the same reduction mechanism as TARP.

There are several limitations in current study. First, the sample size was relatively small. As more cases are performed using this technique, its efficacy may be more thoroughly evaluated. In addition, the present study is retrospective in nature; future prospective studies may better control of follow-up timing intervals and may have the potential to include more standardized outcome measures.

## Conclusions

Revision surgery through transoral intraarticular cage distraction and C-JAWS fixation is an effective procedure in treatment of patients with BI and IAAD after an unsuccessful posterior OCF. This technique can provide satisfactory reduction, fixation and bony fusion, and also offered a new method for transoral atlantoaxial fixation.

## Data Availability

The data used and analyzed during the current study are available in anonymized form from the corresponding author on reasonable request.

## Abbreviations

BI: Basilar invagination
IAAD: Irreducible atlantoaxial dislocation
AAD: Atlantoaxial dislocation
TARP: Transoral atlantoaxial reduction plate
ADI: Atlas-dens interval
CMA: Cervicomedullary angle
CL: Distance between the tip of the dens and Chamberlain line
JOA: Japanese Orthopedic Association score
Chiari: Chiari malformation
KFS: Klippel-Feil syndrome
OCF: Occipitocervical fusion
CT: Computed tomographic
MRI: Magnetic resonance imaging
AA: Atlas assimilation
PRI: Posterior removal of instrument
PFD: Posterior fossa decompression
TAADF: Transoral atlantoaxial distraction and fixation
ACDF: Anterior cervical discectomy and fusion
